# Chromosome-scale, single haploid assembly of achiote (*Bixa orellana*)

**DOI:** 10.1186/s12863-026-01430-w

**Published:** 2026-05-06

**Authors:** Meric C. Lieberman, Margarita Aguilar-Espinosa, David Chan-Rodriguez, Luca Coma, Renata Rivera-Madrid, Isabelle M. Henry

**Affiliations:** 1 UC Davis Department of Plant Biology and Genome Center, University of California, 451 Health Science, Davis, CA 95616 USA; 2 Unidad de Biología Integrativa. Centro de Investigación Científica de Yucatán, A. C, Calle 43 #130 Chuburná de Hidalgo, Mérida, C.P. 97205 Yucatán México; 3 Present Address: Department of Plant Genetics, Breeding and Biotechnology, Institute of Biology, Warsaw University of Life Sciences, Warsaw, Poland

**Keywords:** Genome sequencing, achiote, *Bixa orenalla*, Pigment, Genome assembly

## Abstract

**Objectives:**

*Bixa orellana, *commonly known as achiote, is the domesticated species within the Bixa genus. It is a commercially important plant due to its high bixin content, primarily used as a pigment in the food industry. Achiote is a cross-pollinated, highly heterozygous species with a small genome size. This study aims to generate a de novo genome assembly of *Bixa orellana* L., which will be crucial for identifying the genes involved in bixin synthesis and its regulation. This assembly will offer valuable insights for future applications related to agronomical traits aimed at increasing bixin yield in achiote or different plant or microorganism systems, as well as for improving the genetic potential of achiote crops.

**Data description:**

A de novo assembly of the genome of *Bixa orellana* L., accession PN4 was generated from ~145 x consensus coverage of PacBio long reads. This Hifiasm assembly included 10 chromosome-scale superscaffolds representing 96.4% of the assembly, for an overall genome size of 270 Mb. Illumina RNA-Seq libraries from different tissues sampled from the same cultivar were used to annotate the genome.

## Objective

*Bixa orellana* L., or achiote, is a perennial, woody plant with annual flowering. It is of significant economic importance due to its high bixin content, mainly found in its seeds. *B. orellana* is cross-pollinated and is considered to be a domesticated species of the *Bixa* genus [1]. The main use of bixin is as a dye in the food, pharmaceutical, cosmetics, textile, and paint industries, as well as in the traditional cuisine of several countries. In 2003, Bouvier et al. introduced three genes in *Escherichia coli* as a heterologous expression system to demonstrate that bixin synthesis can be performed using these three genes only [2]. However, the analysis of the achiote transcriptome enabled the identification of several additional genes that may participate in the bixin biosynthetic pathway as well [3, 4]; suggesting that the bixin synthesis pathway is more complex than originally assessed.

Previous cytological investigations in *Bixa orellana* suggested that it harbors a diploid genome with 7 chromosome pairs [5], for a small haploid genome size approaching 200 Mb [6].

Here, we report on the sequencing and assembly of the genome of *Bixa orellana*, accession PN4 [7]. This new genome reference will be used as a blueprint for the analysis of other achiote accessions and functional studies investigating metabolite production in this genus. Likewise, this work will enable faster genetic improvement in Achiote and, eventually, the production of bixin heterologous systems (different plants or microorganisms) for synthesis on a larger scale.

## Data description

A PacBio Hifi read library containing 2,177,607 reads (Table 1, Data set 2) of average length 13.6 kb was assembled using hifiasm in default mode [8]. This resulted in a 270 Mb primary assembly, containing 164 contigs with an N50 of 27.9 Mb (Table 1 Data file 1). Within these contigs were 10 chromosome-scale superscaffolds (Table 1, Data file 2 and Data set 3), all longer than 12 Mb and representing 96.4% of the entire assembly while all other contigs were shorter than 500 kb. This set of 10 superscaffolds represented a total genome size of 261 Mb, with an N50 of 27.4 Mb.

Analysis of 21-mers frequency and GenomeScope profiling [9] suggested ~ 0.76% heterozygosity (Table 1, Data file 9). For the sake of simplicity, we elected to only use the primary assembly in order to build a single set of chromosomes, as opposed to a haplotype-phased genome. Self-mapping of these 10 super scaffolds to each other did not generate any hit of significant length, strongly suggesting that they represent independent chromosomes or chromosome segments, as opposed to haplotypes of each other (Table 1, Data file 8). Comparison of our assembly to the most recent assembly of Cacao (*Theobroma cacao* v2.1) only generated partial matches with our superscaffolds but the same region of the cacao genome did not map to more than one region of our superscaffolds, again suggesting that these represent independent chromosomes (Table 1, Data file 10).

The superscaffolds were analyzed using BUSCO with the eudicots_odb10 dataset [10]. For the entire assembly, the BUSCO yielded 98.6% complete, 2.3% duplicated, 0.3% fragmented, and 1.1% missing, while for the 10 superscaffold set it was 98.5% complete, 1.9% duplicated, 0.3% fragmented, and 1.2% missing. Next, the chromosome scale contigs were annotated using braker3 and RNASeq transcriptome data (Table 1, Data set 2). The RNASeq data was collected from fruit, roots, and flowers, and totaled 127 million PE reads. The reads were quality control processed using trim_galore, merged, and then mapped to the chromosome scaffolds using hisat2 [11]. The mappings were then converted to a bam file for use with braker3 using samtools [12]. Braker3 generated a transcriptome containing 56k transcripts covering a coding space of 64.4 MB (Table 1, Data files 3–5). The resulting transcripts were loaded into Biobam’s Omicsbox [13] for functional mapping to NCBI’s NR database, to the InterPro protein database using InterProScan [14], and to obtain GO annotation mappings [15]. Of the 56k transcripts, 49k generated BLAST hits, and 44.2k were associated with at least one GO functional mapping (Table 1, Data file 6 and 7).

**Table 1:** Overview of data files/data sets

Label	Name of data file/data set	File types (file extension)	Data repository and identifier (DOI or accession number)
*Data file 1*	Full assembled genome (all scaffolds)	*Fasta file (.fa)*	Figshare: 10.6084/m9.figshare.30797783 [16]
*Data file 2*	Assembled genome - chromosome-scale supper-scaffolds only.	*Fast file (.fa)*	Figshare: 10.6084/m9.figshare.30797783 [16]
*Data file 3*	Predicted genes	*Gff3 file (.gff3)*	Figshare: 10.6084/m9.figshare.30797783 [16]
*Data file 4*	Predicted genes – CDS	*Fasta file (.fa)*	Figshare: 10.6084/m9.figshare.30797783 [16]
*Data file 5*	Predicted protein sequence	*Fasta file (.fa)*	Figshare: 10.6084/m9.figshare.30797783 [16]
*Data file 6*	Predicted genomic features	*Gtf file(.gtf)*	Figshare: 10.6084/m9.figshare.30797783 [16]
*Data file 7*	Functional annotation table exported from OmicsBox	*Text file (.txt)*	Figshare: 10.6084/m9.figshare.30797783 [16]
*Data file 8*	Read-me file	*Text file (.txt_*	Figshare: 10.6084/m9.figshare.30797783 [16]
*Data file 9*	Self-mapping of the Achiote supper-scaffolds	*Image file (.png)*	Figshare: 10.6084/m9.figshare.30797783 [16]
*Data file 10*	Mapping of the Achiote supper-scaffolds to the genome of Cacao (*Theobroma cacao)* v2.1.	*Image file (.png)*	Figshare: 10.6084/m9.figshare.30797783 [16]
*Data file 11*	GenomeScope profile of the PacBioHiFi reads using 21-mers.	*Image file (.png)*	Figshare: 10.6084/m9.figshare.30797783 [16]
*Data file 12*	Read-me file	*Text file (.txt)*	Figshare: 10.6084/m9.figshare.30797783 [16]
*Data set 1*	*Raw RNA-seq reads*	*Fastq file (.fq)*	NCBI SRA Accession numbers SRX31347776 to SRX31347783 https://identifiers.org/ncbi/insdc.sra:SRX31347776 [17], https://identifiers.org/ncbi/insdc.sra: SRX31347777 [18], https://identifiers.org/ncbi/insdc.sra: SRX31347778 [19], https://identifiers.org/ncbi/insdc.sra: SRX31347779 [20], https://identifiers.org/ncbi/insdc.sra:SRX31347780 [21], https://identifiers.org/ncbi/insdc.sra: SRX31347781 [22], https://identifiers.org/ncbi/insdc.sra:SRX31347782 [23], https://identifiers.org/ncbi/insdc.sra:SRX31347783 [24]
*Data set 2*	*Raw PacBio reads*	*PacBio basecall format (fq)*	NCBI SRA Accession number SRX31347784 https://identifiers.org/ncbi/insdc.sra:SRX31347784 [25]
*Data set 3*	*Assembled genome*	*Fasta file (.fa)*	NCBI GenBank JBSTUB01 https://identifiers.org/ncbi/insdc:JBSTUB000000000.1 [26]

## Limitations

This annotation was based on 5 transcriptome libraries representing 4 tissue types. It is possible some genes were not represented because they were not highly expressed in the tissues and conditions sampled. We elected to focus on the tissues most likely to express bixin-biosynthetic genes.

Many of the annotated transcripts did not generate functional or GO annotation links. This suggests that these genes are novel or incorrectly annotated. The quality of the mapped annotations therefore ranged from no transcript functional information up to transcripts with protein BLAST hits, GO annotation, and protein functional domain(s).

## Data Availability

The data described in this Data note can be freely and openly accessed at the National Center for Biotechnology Information Short Read Archive and FigShare as described in Table 1. The available datasets are summarized in Table 1. (https://bmcgenomdata.biomedcentral.com/articles/10.1186/s12863-023-01181-y) 1. The *Bixa orellana* accession used in the study, PN4 , can be obtained from the Centro de Investigación Científica de Yucatán Germplasm collection.

## Abbreviations

HiFi: High-fidelity
BUSCO: Benchmarking Universal Single-Copy Orthologs
